# BCMA-CAR-T细胞治疗难治复发性多发性骨髓瘤患者血清脂质水平和营养状态变化及其影响：基于LEGEND-2的回顾性研究

**DOI:** 10.3760/cma.j.issn.0253-2727.2023.10.008

**Published:** 2023-10

**Authors:** 雪珠 许, 睿 刘, 万红 赵, 云 杨, 捷 刘, 王刚 张, 菊 白, 爱丽 何

**Affiliations:** 西安交通大学第二附属医院血液科，西安 710004 Department of Hematopathology, the Second Affiliated Hospital of Xi'an Jiaotong University, Xi'an 710004, China

**Keywords:** 多发性骨髓瘤, CAR-T细胞, 血脂水平, 营养状态, CONUT评分, 预后因素, Multiple myeloma, Chimeric antigen receptor T cell, Serum lipid level, Nutritional status, CONUT score, Prognostic factors

## Abstract

**目的:**

基于LEGEND-2研究探讨BCMA-CAR-T细胞治疗难治复发性多发性骨髓瘤（R/R MM）期间血清脂质水平和营养状态的变化。

**方法:**

以2016年3月至2018年2月在西安交通大学第二附属医院行BCMA-CAR-T细胞治疗的57例R/R MM患者为研究对象，回顾性分析CAR-T治疗前后不同时间点的血清脂质水平、控制营养状况（CONUT）评分等临床指标的变化。通过ROC曲线确定最佳截断值，将患者分为高CONUT评分组（>6.5分，营养状况不良组）和低 CONUT评分组（≤6.5分，营养状况良好组），通过Kaplan-Meier生存分析对比两组的无进展生存（PFS）期及总生存（OS）期。

**结果:**

输注CAR-T细胞前，患者血清脂质水平除甘油三酯（TG）外均低于正常水平。在CAR-T细胞输注后8～14 d内，血清白蛋白（ALB）、总胆固醇（TC）、高密度脂蛋白（HDL）、低密度脂蛋白（LDL）和载脂蛋白A1（Apo A1）水平降至最低，CONUT评分达到最大值，而TG、载脂蛋白B（Apo B）水平较输注前升高。CAR-T细胞治疗后，患者的血清脂质水平显著升高，同时营养状态显著改善。Spearman相关分析显示，CAR-T细胞输注后TC、HDL和Apo A1的水平与细胞因子释放综合征（CRS）分级呈显著负相关（*r*＝−0.548，*P*＝0.003；*r*＝−0.444，*P*＝0.020；*r*＝−0.589，*P*＝0.001）。生存分析提示，输注前的CONUT评分与PFS无关，高CONUT评分组MM 患者较低CONUT评分组患者的中位OS期缩短（*P*＝0.046）。

**结论:**

CAR-T细胞治疗期间患者的血脂水平降低及营养状态不良加剧，这可能与CRS有关；CAR-T细胞治疗后患者血脂水平及营养状态会显著改善。营养状况CONUT评分是影响CAR-T细胞治疗患者OS的因素。

## Abstract (en)

**Objective:**

To explore the dynamic changes in serum lipid levels and nutritional status during BCMA-CAR-T-cell therapy in patients with refractory or relapsed multiple myeloma（R/R MM）based on LEGEND-2.

**Methods:**

The data of patients with R/R MM who underwent BCMA-CAR-T therapy at our hospital between March 30, 2016, and February 6, 2018, were retrospectively collected. Serum lipid levels, controlled nutritional status（CONUT）score, and other clinical indicators at different time points before and after CAR-T-cell infusion were compared and analyzed. The best cut-off value was determined by using the receiver operator characteristic（ROC）curve. The patients were divided into high-CONUT score（>6.5 points, malnutrition group）and low-CONUT score groups（≤6.5 points, good nutrition group）, comparing the progression-free survival（PFS）and total survival（OS）of the two groups using Kaplan-Meier survival analysis.

**Results:**

Before the infusion of CAR-T-cells, excluding triglycerides（TG）, patients’ serum lipid levels were lower than normal on average. At 8-14 d after CAR-T-cell infusion, serum albumin（ALB）, total cholesterol（TC）, high-density lipoprotein（HDL）, low-density lipoprotein（LDL）, and apolipoprotein A1（Apo A1）levels dropped to the minimum, whereas CONUT scores reached the maximum. In addition to TG, apolipoprotein B（Apo B）levels increased compared with baseline. After CAR-T-cell therapy, the patients’ serum lipid levels significantly increased with well-improved nutritional status. Spearman's related analysis showed that TC, HDL, and ApoA1 levels after CAR-T-cell injection were significantly negatively correlated with the grade of cytokine-release syndrome（CRS）（*r*＝−0.548, *P*＝0.003; *r*＝−0.444, *P*＝0.020; *r*＝−0.589, *P*＝0.001）. Furthermore, survival analysis indicated that the CONUT score was unrelated to PFS, and the median OS of patients with R/R MM in the high-CONUT score group was shorter than that in the low-CONUT score group（*P*＝0.046）.

**Conclusion:**

During CAR-T-cell therapy, hypolipidemia and poor nutritional status were aggravated, which is possibly related to CRS. The patients’ serum lipid levels and nutritional status were significantly improved after CAR-T-cell treatment. The CONUT score affected the median OS in patients treated with CAR-T-cells. Therefore, specific screening and intervention for nutritional status in patients receiving CAR-T-cell therapy are required.

多发性骨髓瘤（MM）是一种高度异质性血液系统恶性肿瘤。近年来，免疫调节药物和蛋白酶体抑制剂的联合用药方案，以及诸多新药（如CD38单抗、XPO1抑制剂等）的出现显著延长了患者的总生存（OS）期[1]–[4]。嵌合抗原受体（CAR）T细胞疗法是治疗MM的一种新兴免疫治疗，取得了令人惊喜的疗效[5]–[6]。MM的患者常伴有营养不良及脂质代谢异常，且血清血脂水平随疾病的转归发生变化[7]。但是，目前关于CAR-T细胞治疗期间血脂水平及营养状况变化的研究较少。因此，本研究回顾性分析了57例难治复发性MM（R/R MM）患者接受BCMA-CAR-T细胞治疗期间血脂水平及营养状态的改变。

## 病例与方法

1. 一般资料及CAR-T细胞输注方案：回顾性分析2016年3月至2018年2月西安交通大学第二附属医院血液科BCMA-CAR-T临床试验（NCT03090659）入组的57例R/R MM患者。其中男34例、女23例，中位年龄为54（27～72）岁，IgG型25例、IgA型15例、轻链型17例。全部57例患者在CAR-T细胞输注前均采取单用环磷酰胺处理：环磷酰胺 300 mg·m^−2^·d^−1^，−5～−3 d。抗BCMA CAR-T细胞应用慢病毒转染技术，由2个BCMA靶向单域抗体、4-1BB共刺激分子及CD3ζT细胞活化序列构成。CAR-T细胞分3次输注，每次分别为总剂量的20％、30％和50％，7 d内完成回输。根据体重调整后的患者CAR-T细胞回输剂量的中位数为0.50（0.07～2.10）×10^6^/kg。研究方案经西安交通大学第二附属医院伦理委员会批准（2016002），所有患者均充分知情并签署知情同意书。

2. 临床资料收集、CONUT评分及CRS分级：回顾性分析患者首次输注CAR-T细胞前、输注后1～7 d、8～14 d、15～21 d、22～30 d和31～60 d的淋巴细胞计数绝对值（LYM#）、血清白蛋白（ALB）、甘油三酯（TG）、总胆固醇（TC）、高密度脂蛋白（HDL）、低密度脂蛋白（LDL）、极低密度脂蛋白（VLDL）、载脂蛋白（Apo A1）、载脂蛋白（Apo B）、脂蛋白a（LPa）的水平以及细胞因子白细胞介素-6（IL-6）、肿瘤坏死因子（TNF-α）、白细胞介素-10（IL-10）。脂质相关指标正常范围参考2023年中国血脂管理指南[8]。

CONUT评分由患者血清ALB水平、LYM#和TC水平赋值后求和得出[9]。具体赋值及计算方法见表1。上述3个指标相加得到总分（0～12分），总分越高表示营养状态越差，反之提示营养状况越好。

**表1:** 复发难治性多发性骨髓瘤患者控制营养状况（CONUT）评分计算方法[9]

TC		LYM#		ALB	
参考值（mmol/L）	评分	参考值（×10^9^/L)	评分	参考值（g/L）	评分
<2.59	3	<0.800	3	<25	6
2.59～3.61	2	0.800～1.199	2	25～29	4
3.62～4.66	1	1.200～1.600	1	30～34.9	2
>4.66	0	>1.600	0	35～45	1

注 TC：总胆固醇；LYM#：淋巴细胞计数绝对值；ALB：血清白蛋白

细胞因子释放综合征（CRS）根据Lee等[10]提出的修订的CRS分级系统进行分级。

3. 随访：随访时间截至2022年11月3日，中位随访时间为43.8（0.7～78.1）个月。无进展生存（PFS）期定义为输注靶向BCMA的LCAR-B38M CAR-T细胞之日起至疾病进展、复发或死亡的时间。OS期定义为自输注LCAR-B38M CAR-T细胞之日起至患者死亡或随访终点的时间。

4. 统计学处理：采用SPSS 24.0软件进行数据分析，计量资料以中位数（范围）描述，计数变量以例数（百分比）表示。Brown-Forsythe and Welch ANOVA test用以比较各指标不同时期的变化，采用Games-Howell法对多重比较结果进行校正。Spearman相关性分析评价各项血脂参数、CONUT评分与CRS分级的相关性。生存分析采用Kaplan-Meier方法，Log-rank检验用于生存差异的检验。*P*<0.05为差异有统计学意义。

## 结果

1. 血脂及细胞因子水平变化：如表2所示，输注BCMA-CAR-T细胞前，患者血清脂质除TG外均低于正常范围。输注CAR-T细胞期间，患者的血脂水平变化总体呈现出“先降低后回升”的趋势，具体为：TG、VLDL、LP a在输注后1～7 d达到最低水平，TC、HDL、LDL、Apo A1在输注后8～14 d达到最低水平，而Apo B在输注后2周内未见明显变化。CAR-T细胞输注后15～21 d，TG和VLDL达到最高水平且显著高于输注前（*P*<0.05），而Apo B、TC、LDL和HDL水平于输注后22～30 d达到最高水平，而Apo A1在输注后22～30 d时仍有上升趋势。

**表2:** 患者输注BCMA CAR-T细胞治疗前后不同时间点血脂及细胞因子水平变化

项目	输注前	输注后1～7 d	输注后8～14 d	输注后15～21 d	输注后22～30 d	输注后31～60 d
TC[mmol/L，*M*（范围）]	3.30（0.85～5.53）	2.66（1.63～4.73）	2.62（1.36～5.55）	3.29（1.65～6.41）	4.14（2.46～7.86）^b,c,e^	3.79（1.20～6.64）^a,b,c^
TG[mmol/L，*M*（范围）]	1.85（0.26～3.97）	0.96（0.50～2.39）^e^	1.47（0.83～3.86）	2.04（0.83～8.07）^a,b^	1.84（0.71～4.66）	1.54（0.44～3.65）
HDL[mmol/L，*M*（范围）]	0.80（0.30～1.70）	0.80（0.36～1.37）	0.62（0.19～1.19）^a^	0.65（0.37～0.96）^a^	0.90（0.54～1.56）^c,d^	0.83（0.50～1.74）^c,d^
LDL[mmol/L，*M*（范围）]	1.70（0.23～3.95）	1.55（0.55～2.89）	1.32（0.51～3.99）	1.94（0.81～4.64）^b^	2.44（1.15～5.48）^b,e^	2.34（0.24～4.66）^b^
VLDL[mmol/L，*M*（范围）]	0.45（0.04～1.30）	0.43（0.12～0.91）	0.49（0.05～1.31）	0.76（0.28～2.22）^b,e^	0.76（0.12～2.25）^b,e^	0.66（0.17～1.40）^b^
Apo A1[g/L，*M*（范围）]	0.90（0.40～1.67）	0.88（0.47～1.31）	0.75（0.38～1.08）^a^	0.84（0.54～1.14）	1.01（0.53～2.07）	1.09（0.50～1.75）^a,b,c,d^
Apo B[g/L，*M*（范围）]	0.59（0.10～1.49）	0.55（0.31～1.31）	0.61（0.25～1.54）	0.75（0.40～1.75）^a,e^	0.80（0.56～1.64）^a,e^	0.76（0.14～1.57）^e^
LP a[g/L，*M*（范围）]	10.40（0.50～72.30）	8.60（1.90～55.10）	9.20（1.90～37.77）	12.15（0.50～35.05）	17.77（2.70～65.50）	14.00（0.50～64.00）
IL-6[ng/L，*M*（范围）]	2.22（2.00～59.40）	3.92（2.00～359.70）	31.45（2.49～793.25）^a,b^	24.40（2.00～479.30）^c^	17.15（2.00～554.00）^c^	9.10（2.00～833.33）^c^
IL-10[ng/L，*M*（范围）]	5.00（5.00～25.70）	5.47（5.00～81.25）	91.14（6.18～1000.00）^a,b^	5.82（5.00～85.20）^c^	5.00（5.00～411.33）^c^	5.00（5.00～64.67）^c^
TNF-α[ng/L，*M*（范围）]	11.10（4.00～131.00）	13.35（6.27～126.00）	23.98（6.33～186.85）^a,b^	15.10（4.69～55.80）^c^	9.83（5.02～87.73）^c^	10.50（5.02～25.44）^c^

注 TC：总胆固醇；TG：甘油三酯；HDL：高密度脂蛋白；LDL：低密度脂蛋白；VLDL：极低密度脂蛋白；Apo A1：载脂蛋白A1；Apo B：载脂蛋白B；LP a：脂蛋白a；IL-6：白细胞介素-6；IL-10：白细胞介素-10；TNF-α：肿瘤坏死因子-α；与输注前相比，^a^*P*<0.05；与输注后1～7 d相比，^b^*P*<0.05；与输注后8～14 d相比，^c^*P*<0.05；与输注后15～21 d相比，^d^*P*<0.05；与正常范围相比，^e^*P*>0.05

此外，输注CAR-T细胞后，患者的IL-6、TNF-α、IL-10水平变化总体呈现出“先升高后降低”的趋势，其达峰时间均为输注后8～14 d，与TC、HDL、LDL、Apo A1达到最低点的时间一致。

2. CONUT评分变化：输注BCMA-CAR-T细胞前，患者的中位CONUT评分为6（2～12）。输注后，CONUT评分表现为“先升高再下降”，于输注后8～14 d达到最高，随后迅速降低，在输注后31～60 d CONUT评分达到最低，并显著低于输注前（*P*<0.05），见表3。

**表3:** 患者输注BCMA-CAR-T细胞治疗前后不同时间点控制营养状况评分变化

项目	输注前	输注后1～7 d	输注后8～14 d	输注后15～21 d	输注后22～30 d	输注后31～60 d
TC[mmol/L，*M*（范围）]	3.30（0.85～5.53）	2.66（1.63～4.73）	2.62（1.36～5.55）	3.29（1.65～6.41）	4.14（2.46～7.86）^b,c^	3.79（1.2～6.64）^a,b,c^
LYM#[×10^9^/L，*M*（范围）]	1.09（0.36～4.42）	0.68（0.21～2.61）	0.92（0.24～6.42）	1.55（0.51～6.45）	1.10（0.04～6.39）	1.14（0.29～5.39）
ALB[g/L，*M*（范围）]	35.95（20.30～48.50）	36.65（21.30～45.20）	34.60（16.95～41.50）	36.30（27.20～41.50）	41.03（30.60～51.00）^c^	43.05（30.60～54.00）^a,b,c,d^
CONUT评分[分，*M*（范围）]	6（2～12）	6.5（1～10.5）	7（1.5～10）	4.5（1.5～9）	4.5（1～9）	4（1～10）^b,c^

注 TC：总胆固醇；LYM#：淋巴细胞计数绝对值；ALB：白蛋白；CONUT：控制营养状况；与输注前相比，^a^*P*<0.05；与输注后1～7 d相比，^b^*P*<0.05；与输注后8～14 d相比，^c^*P*<0.05；与输注后15～21 d相比，^d^*P*<0.05；与正常范围相比，^e^*P*>0.05

3. 血脂水平和营养状态变化与CRS的关系：本研究中共有51例患者（91％）在CAR-T细胞输注后发生CRS。以1级（27/56, 48％）或2级（20/56, 36％）为主，仅4例患者（7％）发生了3级CRS。为了探讨CRS与血脂水平、营养状态之间的关系，我们分析了不同CRS分级患者的各项血脂参数水平（表4），Spearman相关性分析结果显示：CRS分级与输注后8～14 d的TC（*r*＝−0.547）、HDL（*r*＝−0.444）、Apo A1（*r*＝−0.589）呈负相关，与输注后8～14 d的CONUT评分（*r*＝0.464）、15～21 d的TG（*r*＝0.514）、LPa（*r*＝0.459）呈正相关，提示CRS严重程度与患者血脂和营养状态变化密切相关。

**表4:** 血脂水平、控制营养状况评分与细胞因子释放综合征的相关性分析结果

项目	时间	*r*值	*P*值
TC	输注前	0.073	0.592
	输注后1～7 d	0.029	0.903
	输注后8～14 d	−0.547	0.003
	输注后15～21 d	−0.017	0.942
VLDL	输注前	0.208	0.124
	输注后1～7 d	0.332	0.152
	输注后8～14 d	0.180	0.368
	输注后15～21 d	0.304	0.181
HDL	输注前	−0.043	0.755
	输注后1～7 d	0.002	0.992
	输注后8～14 d	−0.444	0.020
	输注后15～21 d	−0.357	0.112
Apo B	输注前	−0.030	0.824
	输注后1～7 d	−0.034	0.887
	输注后8～14 d	0.032	0.873
	输注后15～21 d	0.225	0.326
ALB	输注前	0.028	0.835
	输注后1～7 d	−0.044	0.852
	输注后8～14 d	−0.490	0.010
	输注后15～21 d	−0.210	0.360
TG	输注前	0.002	0.990
	输注后1～7 d	0.347	0.134
	输注后8～14 d	0.237	0.235
	输注后15～21 d	0.514	0.017
Apo A1	输注前	−0.111	0.414
	输注后1～7 d	−0.046	0.847
	输注后8～14 d	−0.589	0.001
	输注后15～21 d	−0.363	0.105
LDL	输注前	0.014	0.916
	输注后1～7 d	0.048	0.842
	输注后8～14 d	−0.223	0.264
	输注后15～21 d	−0.085	0.714
LP a	输注前	0.084	0.539
	输注后1～7 d	0.151	0.526
	输注后8～14 d	0.129	0.529
	输注后15～21 d	0.459	0.036
CONUT评分	输注前	0.051	0.708
	输注后1～7 d	0.001	0.997
	输注后8～14 d	0.464	0.015
	输注后15～21 d	0.253	0.269

注 TC：总胆固醇；VLDL：极低密度脂蛋白；HDL：高密度脂蛋白；Apo B：载脂蛋白B；ALB：白蛋白；TG：甘油三酯；Apo A1：载脂蛋白A1；LDL：低密度脂蛋白；LP a：脂蛋白a；CONUT：控制营养状况

4. CONUT评分对CAR-T细胞疗效及患者生存的影响：我们首先根据受试者工作特征（ROC）曲线确定Youden指数（定义为敏感度＋特异度−1）最大值对应的CONUT评分为6.5，以此作为截断点将患者分为高CONUT评分组（>6.5分）和低CONUT评分组（≤6.5分）。高CONUT评分组19例患者，完全缓解率（CRR）为57.9％，低CONUT评分组CRR为73.7％，两组间的CRR差异无统计学意义（*P*＝0.270）。57例患者的中位PFS期为18.9（95％ *CI* 9.4～25.8）个月。高CONUT评分组和低CONUT评分组的中位PFS期分别为17.6（95％ *CI* 3.6～31.6）个月和20.7（95％ *CI* 8.8～32.7）个月，差异无统计学意义（*P*＝0.884）（图1）。随后，我们进行Kaplan-Meier生存分析，结果显示两组相比，高CONUT评分组患者的中位OS期均显著短于低CONUT评分组（*P*＝0.046）（图1）。

**图1:**
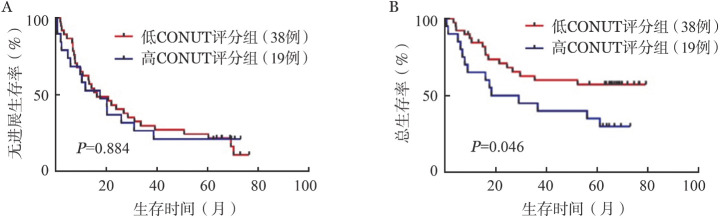
高控制营养状况（CONUT）评分组与低CONUT评分组患者的无进展生存曲线（A）、总生存曲线（B）

5. CAR-T细胞治疗对患者营养状态的改善：以CAR-T细胞输注后第21天为截点，分为CAR-T治疗中和CAR-T治疗后，分析CAR-T治疗前、中、后患者营养状态的改善情况（表5）。结果显示，CAR-T治疗中，高CONUT评分组例数较治疗前增加；CAR-T治疗后，高CONUT评分组例数较治疗前减少，差异具有统计学意义，表明CAR-T细胞治疗可显著改善患者的营养状态。

**表5:** CAR-T治疗对患者控制营养状况（CONUT）评分的影响［例（%）］

组别	CAR-T治疗前	CAR-T治疗中	CAR-T治疗后
CONUT≥6.5	19（33.9）^a,b^	21（61.8）	4（9.8）
CONUT<6.5	37（66.1）^a,b^	13（38.2）	37（90.2）

合计	56	34	41
*P*值		<0.0001

注 与CAR-T治疗中相比，^a^*P*<0.05；与CAR-T治疗后相比，^b^*P*<0.05

## 讨论

MM是一种浆细胞异常增殖、分泌过量单克隆免疫球蛋白的血液系统恶性肿瘤。陆续有研究发现，部分MM患者体内存在血脂水平及营养状况的异常，二者与肿瘤的发生、发展密切相关，血清脂质和白蛋白含量可预示肿瘤预后。近十年来，CAR-T细胞疗法在治疗血液系统恶性肿瘤方面取得了长足的进步, 特别是在治疗R/R MM方面显示出巨大的前景[11]–[12]，但其潜在毒性和不良反应也开始逐步受到研究人员的关注[13]。

目前，关于CAR-T治疗对多发性骨髓瘤患者血脂水平和营养状态影响的研究较少。本研究回顾性分析了接受BCMA-CAR-T治疗R/R MM患者的营养状况变化。研究结果显示，TC、HDL、LDL和Apo A1在CAR-T细胞输注后8～14 d降至最低水平，而CONUT评分达到最高，同时TG、Apo B开始回升。这种现象可能与BCMA-CAR-T细胞治疗后导致的CRS有关。

既往研究表明，MM患者血清中TC、LDL、HDL和Apo A1水平显著降低[14]–[15]，而TG水平升高，且随着疾病进展进一步改变，这与我们CAR-T细胞输注前的结果一致。这可能是由于骨髓瘤细胞对胆固醇的利用增加，高度增殖的肿瘤细胞需要摄取更多外源性胆固醇用来合成细胞膜。

在本研究中，在CAR-T细胞输注后8～14 d内患者血清ALB、TC、HDL、LDL、Apo A1达到最低水平，TG在缓慢回升，而CONUT评分达到最高。既往研究表明CRS多发生于输注后7～14 d[16]，于是我们分析了血脂水平变化与CRS的关系。Spearman相关性分析显示血清ALB、TC、HDL、Apo A1与CRS分级呈负相关，而TG和CONUT评分与CRS分级呈正相关。CRS是输注的CAR-T细胞激活免疫细胞及内皮细胞，由此导致大量细胞因子的释放，如IL-6、TNF-α、IL-10[17]。细胞因子对脂质代谢有深远的影响。细胞因子（如IL-6）可导致白蛋白水平减低[18]，外源性给予TNF-α可以通过刺激肝脏合成和分泌TG[19]，而血浆TNF-α水平与血浆高密度脂蛋白胆固醇（HDL-C）则呈负相关[20]。Apo A1是HDL-C的主要蛋白质成分，因此，Apo A1变化趋势与HDL一致，在炎症反应中呈现低水平状态。Moraitis 等研究表明[21]，内源性IL-10升高能够导致HDL、LDL水平降低，TG水平升高。这一发现也部分解释了当机体处于炎症反应最剧烈的时候（输注后8～14 d），TG、Apo B水平开始缓慢回升。此外，当机体处于强烈氧化应激状态下，通过促进葡萄糖氧化分解过程、增加急性期反应蛋白合成、抑制脂肪酸摄取、提高基础代谢率等，也可能对血清脂质水平产生影响[22]。

CONUT评分是结合了TC、ALB和LYM#的一种简单、客观、经济、便于测量的免疫营养指标[23]。自2005年被首次提出以来，许多研究相继证实，该营养状态评分与恶性肿瘤患者的预后密切相关[24]。有研究指出高CONUT评分是影响MM患者预后的危险因素[25]，这与我们研究发现的CONUT评分对接受BCMA-CAR-T治疗的MM患者的预后有显著影响的结果一致。然而，患者入组CAR-T后，若出现疾病进展，可能会接受包括CAR-T在内的其他治疗，因此，CONUT评分与OS的相关性并不完全与第一次CAR-T的疗效相关。探究CONUT评分与短期评价指标如PFS、缓解率的关系或许能更好地反映第一次CAR-T的疗效。本研究尚未发现CONUT评分与缓解率、PFS之间存在明显相关性，这与既往研究报道[25]不一致，可能是样本存在偏倚或样本量较少。ALB和TC一方面是评估营养状况和肝脏合成能力的参数，另一方面能够反映机体的炎症状态和肿瘤患者的预后。因此，营养状况在血液系统恶性肿瘤患者的治疗反应和生存中起着重要作用。营养不良会降低患者对化疗的耐受性，并增加继发感染的风险。然而，CONUT评分与血液系统恶性疾病的临床结局关系的机制尚未明确，目前关于CONUT评分的截断值也尚未统一。因此，我们建议在血液系统恶性疾病诊疗中，增加对患者营养状态的监测和评估，通过多中心临床研究获得更可信的证据，以改善患者的临床结局和生存质量。

综上所述，R/R MM在CAR-T细胞治疗后不同营养相关生化参数的改变存在差异。血清脂质水平的变化可能与CRS有关，CONUT评分与CRS呈正相关，且CONUT评分对CAR-T细胞治疗后患者的预后有显著影响。在未来临床研究中，需要探索接受CAR-T细胞治疗的患者营养不良的具体筛查和干预措施。
